# Supplementary material for: Investigation of an acute neurological outbreak in Eluru, India, 2020
Source: PLoS One. 2021 Nov 8;16(11):e0259192. doi: 10.1371/journal.pone.0259192 (PMC8575185; doi:10.1371/journal.pone.0259192)
Supplement: Attachment — Submitted filename: Responce to Reviewers.docx [file pone.0259192.s005.docx]

To,

The Editor in Chief,

PLOS ONE

Sub: Submission of Revised manuscript of original research paper titled “**Investigation of an Acute Neurological Outbreak in an Urban town of South India**” – reg;

Sir,

We are pleased to get an initial remark from your reviewers.

Hereby we submit the corrections made accordingly.

**Question 1.** Please ensure that your manuscript meets PLOS ONE's style requirements, including those for file naming. The PLOS ONE style templates can be found at

<https://journals.plos.org/plosone/s/file?id=wjVg/PLOSOne_formatting_sample_main_body.pdf> and <https://journals.plos.org/plosone/s/file?id=ba62/PLOSOne_formatting_sample_title_authors_affiliations.pdf>

**Answer 1:** we have revised our manuscript as per the PLOS ONE’s style requirement

**Question 2.** In your Methods section, please provide additional information regarding the permits you obtained for the work. Please ensure you have included the full name of the authority that approved the field site access and, if no permits were required, a brief statement explaining why.

**Answer 2:** we have included a paragraph “Permission to conduct the study (2.7.1)”.

3. Thank you for including your ethics statement: "Ethical Clearance was obtained from the ICMR-NIN Institutional Ethical Committee. IEC Registration Number- ECR/35/Inst/AP/2013, Study Protocol Number-1/I/2021. Informed written consent

was taken from the study participants to obtain the samples after explaining the procedures. ". Please amend your current ethics statement to include the full name of the ethics committee/institutional review board(s) that approved your specific study.

Once you have amended this/these statement(s) in the Methods section of the manuscript, please add the same text to the “Ethics Statement” field of the submission form (via “Edit Submission”).

For additional information about PLOS ONE ethical requirements for human subjects research, please refer to <http://journals.plos.org/plosone/s/submission-guidelines#loc-human-subjects-research>.

**Answer 3:** we have included full name of the ethics committee/institutional review board that approved the present study.

4. We note that you have indicated that data from this study are available upon request. PLOS only allows data to be available upon request if there are legal or ethical restrictions on sharing data publicly. For information on unacceptable data access restrictions, please see <http://journals.plos.org/plosone/s/data-availability#loc-unacceptable-data-accessrestrictions>.

a) If there are ethical or legal restrictions on sharing a de-identified data set, please explain them in detail (e.g., data contain potentially identifying or sensitive patient information) and who has imposed them (e.g., an ethics committee). Please also provide contact information for a data access committee, ethics committee, or other institutional body to which data requests may be sent.

b) If there are no restrictions, please upload the minimal anonymized data set necessary to replicate your study findings as either Supporting Information files or to a stable, public repository and provide us with the relevant URLs, DOIs, or accession numbers. Please see http://www.bmj.com/content/340/bmj.c181.long for guidelines on how to de-identify and

prepare clinical data for publication. For a list of acceptable repositories, please see <http://journals.plos.org/plosone/s/data-availability#loc-recommended-repositories>.

We will update your Data Availability statement on your behalf to reflect the information you provide.

Answer 4: we have no restriction in sharing de-identified data set. We have uploaded as supporting Information files

**5. Review Comments to the Author**

Please use the space provided to explain your answers to the questions above. You may also include additional comments for the author, including concerns about dual publication, research ethics, or publication ethics. (Please upload your review as an attachment if it exceeds 20,000 characters)

**Reviewer #1:**

1) Biological samples collection and water collection should be detailed described.

A: As suggested, we have included the methodology of blood, urine and water sample collection.

2) Sample preparation for the analysis should be described.

A: As suggested, we have included sample preparation for the analysis

3) Is there any data about cholinesterase activity of the patients?

A: As we have found organophosphorus in both blood and its metabolites in urine samples of the cases, which was confirmed by high resolution LC-M/S, hence we haven’t analysed samples for any cholinesterase activity.

4) Is there any limit for triazophos residue in food?

A: There is No minimum limit for Triazophos residue in food.

**Reviewer #2:**

1) In the methods section: it is necessary to better describe the analysis of pesticide detection. I see that it is necessary to put important information as an extractor solvent and which adsorbent used.

A: As suggested, we have included the methodology of sample preparation with detailed methodology of pesticide analysis

2) In the results section: The presentation of the data is very confusing and would indicate modifying the types of graphs presented. Including that screen prints are rarely used in a scientific article.

A: As suggested, we have included figures and table for better presentation of data. We have removed screen prints and uploaded images in high resolution (PACE approved)

3) In the discussion session: I think it is necessary to place the limitations of the study in the initial part of the discussion.

A: As suggested, we have kept the limitations of the study in earlier paragraph of discussion.

We hope, we have submitted the suggested corrections and ready for more if any. Thank you for consideration.

Yours sincerely,

Sinha Sukesh Narayan, PhD.
Scientist – F, Div. of Food Safety,
ICMR – National Institute of Nutrition,
Tarnaka, Hyderabad – 500007.
