# Supplementary material for: Investigation of an acute neurological outbreak in Eluru, India, 2020
Source: PLoS One. 2021 Nov 8;16(11):e0259192. doi: 10.1371/journal.pone.0259192 (PMC8575185; doi:10.1371/journal.pone.0259192)
Supplement: Attachment — Submitted filename: Responce to Reviewers.docx [file pone.0259192.s006.docx]

To,

The Editor in Chief,

PLOS ONE

Sub: Submission of **Second Revised manuscript** of original research paper titled “**Investigation of an Acute Neurological Outbreak in an Urban town of South India**” – reg;

Sir,

We are really grateful to the editorial board and reviewers for reviewing our study. Hereby we submit the corrections made accordingly and sending second revised manuscript.

**1.** Please include captions for your Supporting Information files at the end of your manuscript, and update any in-text citations to match accordingly. Please see our Supporting Information guidelines for more information: <http://journals.plos.org/plosone/s/supporting-information>.

**Answer:** we have included captions for supporting information as per the guidelines.

**Reviewer #1:**

**Question:** The manuscript was improved after the revision. All my comments were addressed; therefore, the manuscript could be accepted for publication.

**Answer:** Thank you for your kind suggestions.

**Reviewer #2:**

**Question 1:** The abstract is very large and divided into two parts. I suggest that the abstract be reduced to 1 page and the reduction occurs mainly in the introduction to the subject.

**Answer1:** Abstract was reduced to one page as per the suggestions.

**Question 2:** Introduction: I found the introduction to this type of article with proposed design unusual. I suggest you improve the introduction by discussing more general outbreak cases, because when treated in this way it looks like an article of regional interest.

**Answer 2:** We have added general outbreak cases in introduction as suggested.

**Question 3:** Methodology: I am in doubt whether this would actually be an exploratory cross-sectional study or a case-control study. I believe this study is a case-control, where the design is widely used in cases of outbreaks. Most case-control studies use markers to identify the exposure that caused the effect. Please review your study design and if you stick to your decision, I expect plausible arguments for that decision.

**Answer 3:** After a thorough internal discussion, we have reviewed the study design and appropriately have corrected the design as case-control study. Thank you for the kind suggestion.

**Question 4:** Results: It's good

**Answer 4:** Thank you.

**Question 5:** Discussion: I think it is necessary to talk more about toxicological studies related to exposure to a variety of contaminants and the adverse effects that can be observed.

**Answer 5:** As suggested, we have discussed few toxicological studies with health effects related to variety of contaminants.

**Question 6:** Conclusion: It is not a conclusion of the study to say that this is the first study of the outbreak. The conclusion needs to be made from the main findings found and indicate solutions or possible ways forward.

**Answer 6:** The conclusion is rewritten by mentioning main findings of the study and related solutions as suggested.

Respected Reviewers, kindly go through the second revised manuscript with above corrections made as per your kind suggestions and do the needful.

Yours sincerely,

Sinha Sukesh Narayan, PhD.
Scientist – F, Div. of Food Safety,
ICMR – National Institute of Nutrition,
Tarnaka, Hyderabad – 500007.
